# Goslin 2.0 Implements the Recent Lipid Shorthand Nomenclature for MS-Derived Lipid Structures

**DOI:** 10.1021/acs.analchem.1c05430

**Published:** 2022-04-11

**Authors:** Dominik Kopczynski, Nils Hoffmann, Bing Peng, Gerhard Liebisch, Friedrich Spener, Robert Ahrends

**Affiliations:** † Institute of Analytical Chemistry, University of Vienna, 1090 Vienna, Austria; ‡ Center for Biotechnology (CeBiTec), Bielefeld University, 33594 Bielefeld, Germany; ∥ Division of Rheumatology, Department of Medicine, Solna, Karolinska Institutet and Karolinska University Hospital, 17176 Stockholm, Sweden; ⊥ Institute of Clinical Chemistry and Laboratory Medicine, Regensburg University Hospital, 93053 Regensburg, Germany; ○ Department of Molecular Biosciences, University of Graz, 8010 Graz, Austria; ◇ Division of Molecular Biology and Biochemistry, Gottfried Schatz Research Center, Medical University of Graz, 8036 Graz, Austria

## Abstract

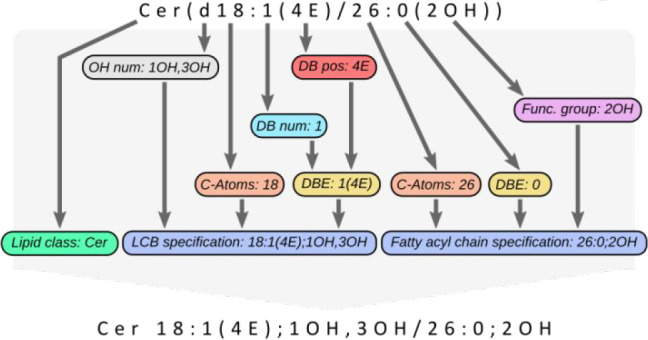

Goslin is the first grammar-based computational library for the recognition/parsing and normalization of lipid names following the hierarchical lipid shorthand nomenclature. The new version Goslin 2.0 implements the latest nomenclature and adds an additional grammar to recognize systematic IUPAC-IUB fatty acyl names as stored, e.g., in the LIPID MAPS database and is perfectly suited to update lipid names in LIPID MAPS or HMDB databases to the latest nomenclature. Goslin 2.0 is available as a standalone web application with a REST API as well as C++, C#, Java, Python 3, and R libraries. Importantly, it can be easily included in lipidomics tools and scripts providing direct access to translation functions. All implementations are open source.

## Introduction

Lipids are essential organic molecules, since they are responsible for the compartmentalization of cells by membrane formation for the storage of energy and for serving as signaling molecules. No other molecule class comprises all these characteristics. Lipidomics is the research field based on large-scale lipid characterization by bioanalytical methods combined with data analysis by bioinformatics and the interpretation of these data in a broader biological context. Thereby, mass spectrometry (MS) drives the field due to its speed, sensitivity, and specificity.^1,2^ On the one hand, this field is highly dependent on computational approaches, providing researchers with fast and accurate algorithms for conducting MS-based lipidomics experiments due to the complexity of lipid structures and lipidomes. On the other hand, hierarchical shorthand descriptions of lipids were introduced by Liebisch et al.^3,4^ to concisely represent the vast structural variety of lipids and to thereby enable consistent reporting and communication. Recently, this shorthand nomenclature was refined and extended^3^ to support more lipid classes and specific lipids with new features. The main updates were the annotation of ring double bond equivalents instead of double bonds and the number of oxygen atoms to permit a hierarchical reporting of oxygenated lipid species. The fatty acyl category was completely updated and now covers fatty acids and conjugates, fatty alcohols, wax monoesters, N-acyl amines, etc. In addition, shorthand notations for functional groups were added to the nomenclature, such as the COOH and OOH groups. Enclosed structures like acyl/alkyl branches or cycles can now be described as well. The hierarchical description levels representing the structural knowledge about a lipid were also updated as shown in Table 1. These changes will require updates of existing lipidomics tools and databases.

**Table 1:** Hierarchical Presentation of a Shorthand Notation for Oxygenated Phosphatidylethanolamine PE 16:1(6*Z*)/16:0;5OH[*R*],8OH[*S*];3oxo^a^

level	lipid name
category	GP
class	PE
species level	PE 32:2;O3
molecular species level	PE 16:1_16:1;O3
*sn*-position level	PE 16:1/16:1;O3
structure defined level	PE 16:1(6)/16:0;(OH)2;oxo
full structure level	PE 16:1(6*Z*)/16:0;5OH,8OH;3oxo
complete structure level	PE 16:1(6*Z*)/16:0;5OH[*R*],8OH[*S*];3oxo

a From top to bottom, the structural information of the molecule increases. The species level provides information about the head group plus aggregated information on fatty acyl chains. The molecular species level provides aggregated information about constituent fatty acyl chains with unknown *sn*-positions. The *sn*-position level clarifies stereo-specific numbering. Until this level, the double bonds in the functional groups may be aggregated in the double bond equivalent. The structure defined level resolves functional groups in constituent fatty acyl chains. The full structure level adds position information, while the complete structure level adds all stereo-chemical information.

Besides this shorthand notation, several other systematic nomenclatures for lipids exist. In a linguistic context, these nomenclatures can be considered languages to describe how lipid names have to be structured. Since these nomenclatures are closely related and produce similar lipid names, we denote them as dialects. It remains a challenging task to correctly identify a lipid by its name among all the different dialects. The lipidomics field currently faces the challenges of integration and reanalysis of the existing results and data sets from multiple tools and data repositories that use different lipid shorthand dialects in order to document and reproduce lipid identification and quantification results. Making lipidomics research data machine-readable and accessible via community-accepted data formats, common shorthand names that encode the structural knowledge of lipids is one of the first steps required to address those challenges in accordance with the mission of the FAIR principles of interoperability and reusability.^5^

In 2020, we introduced Goslin^6^ (the “Grammar Of Succinct LIpid Nomenclature”), which is a framework to translate lipid names from different dialects into a standardized name according to the lipid shorthand nomenclature. Its key module is a so-called parser that, based on a dialect-specific formal grammar, disaggregates the input string (i.e., lipid name), checks for correct syntax, and interprets these fragments to generate a standardized lipid name. Each grammar is a set of rules on what valid lipid names must look like according to the nomenclature. RefMet^7^ is a database and web application that supports the normalization of names in metabolomics data with support for lipidomics shorthand nomenclature. LipidLynxX^8^ provides a web application to normalize lipid names in order to interlink lipidomics data sets with external data sources, e.g., in the context of integration with biological pathways.

Goslin is already well accepted in the community and utilized in different tools such as LipidSuite, Lux Score 2.0, or lipidomics workflows.^9−11^ To allow quick adaptation of the computational field and to keep the framework up to date and usable, we released the new version Goslin 2.0 with new features and full support of the latest shorthand nomenclature.

## Methods

Goslin can be used directly as a web application with an HTML user interface supplemented by a REST API for computational access at https://apps.lifs-tools.org/goslin/. In addition, it is available as a library for the programming languages C++, C#, Java, Python, and R. Programmers can easily include these libraries in their tools and use the provided lipid name translation functions directly. Here, we present the updated version Goslin 2.0 with several new features with an updated set of lipid classes (see Table S1).

**Figure 1:**
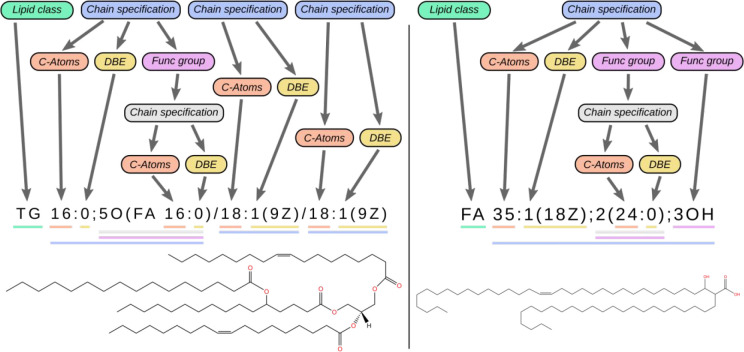
Exemplary illustrations of nested patterns: left, triacylglycerol with additional O-acyl linkage;^3^ right, branched fatty acyl chain (LIPID MAPS-ID LMFA01160041) can be aligned schematically into the substitution blocks [*lipid class*] and [*chain specification*]. Here, the blocks [*chain specification*] (blue) are substituted into their successors. Some [*functional group*] blocks are again substituted into a [*chain specification*] block (gray) to describe the attached fatty acyl (left) or alkyl (right) branches in their lipids. A chain specification appears within another chain specification (gray within blue).

Goslin fully supports the new shorthand nomenclature and is, to the best of our knowledge, the only tool capable of handling nested or recursive structures. For example, a typical lipid notation has the following structure: “[*lipid class*] [*chain specification*]/[···]”, e.g., “TG 16:0/18:1(9*Z*)/18:1(9*Z*)”. The *chain specification* itself usually has the structure “[*C-atoms number*]:[*double bond equivalent*];[*functional group 1*];[*functional group 2*];[···]”, e.g., “16:0;3OH”, where all functional groups are added sequentially at the end and are separated by a semicolon. Usual functional groups are, for instance, hydroxy groups (OH), carboxy groups (COOH), or sometimes whole O-acyl branches such as for TG 16:0;5O(FA 16:0)/18:1(9*Z*)/18:1(9*Z*). Here, [*functional group*] corresponds to the pattern [*chain specification*]. The *specification* of the O-acyl branch “O(FA 16:0)” is enclosed as a functional group at the carbon 5 position within the complete *specification* of the first fatty acyl chain “16:0;5O(FA 16:0)”. An illustration of this nested pattern is provided in Figure 1. We use context-free grammars and parsers, which can recognize these kinds of patterns. According to the Chomsky hierarchy, standard approaches utilizing regular expressions do not have the possibility of recognizing general nested patterns as formally correct^12^ and thus are insufficient for our purposes. Incorrect recognition of lipid names (and thus no or incorrect error handling) results in incorrect annotations.

Another new feature is the additional grammar that parses systematic fatty acyl descriptions following the IUPAC-IUB nomenclature,^13^ such as that listed in Table 2. This feature enables databases with older entries to convert their fatty acyl IUPAC-IUB names into the newest lipid nomenclature. The third major new feature of Goslin 2.0 is the calculation of the chemical sum formula of lipids and accurate masses (neutral or adduct ions). As in the preceding version, the user can choose at which level the lipid annotation should be generated in accordance with the hierarchy shown in Table 1. All information that can be extracted from a lipid shorthand notation can be obtained from Goslin 2.0 either as a table from the web service at https://apps.lifs-tools.org/goslin/ or as the associated data structures from the programming libraries. Supported lipid dialects are the updated shorthand notations by Liebisch et al.,^3^ the original shorthand notation by Liebisch,^4^ i.e., LIPID MAPS dialect,^14^ Goslin dialect, SwissLipids^15^ dialect, HMDB^16^ dialect, and the IUPAC-IUB nomenclature dialect for fatty acyl chains.

**Table 2:** Examples for Lipid Naming by IUPAC-IUB and Standardization by Shorthand Notation

IUPAC-IUB name	LIPID MAPS	standardized name
5-methyl-octadecanoic acid	LMFA01020216	FA 18:0;5Me
2-docosyl-3-hydroxy-28,29-epoxy-30-methyl-pentacontanoic acid	LMFA01160100	FA 50:0;2(22:0);30Me;28Ep;3OH
11*R*-hydroxy-9,15-dioxo-2,3,4,5-tetranor-prostan-1,20-dioic acid	LMFA03010032	FA 15:0;[4-8cy5:0;7OH;5oxo];11oxo;15COOH
*N*-((±)-8,9-dihydroxy-5*Z*,11*Z*,14*Z*-eicosatrienoyl)-ethanolamine	LMFA08040030	NAE 20:3(5*Z*,11*Z*,14*Z*);8OH,9OH

## Results

We evaluated Goslin 2.0 by taking all fatty acyl chain descriptions from LIPID MAPS along with their chemical sum formula and converting them via Goslin. The LIPID MAPS database contains 10 011 fatty acyls (July 2021). From this set, 8005 lipid names can be specified with the new nomenclature. The remaining entries contain structures such as triple bonds, histidine, aspartate, azaniumyl, etc. that cannot be described by the nomenclature yet. The conversion and computation of the chemical sum formula of all lipid names took less than 3 s (when applying the C++ library) on our standard computing platform (Lenovo Thinkpad X1 Carbon, Intel i7 1.8 GHz octa-core laptop, 16GB main memory). All computed sum formulas did perfectly match with the sum formulas from the database. Additionally, we randomly picked lipid names from the translated list and manually checked their correctness according to the nomenclature specifications. All lipid names were correct. We updated our previous unit tests (>100 000 single tests) to the new nomenclature. All tests passed without problems on each system (C++/C#/Java/Python/R). To check the overall performance of Goslin 2.0, we took the databases from LIPID MAPS, SwissLipids, and HMDB and selected fatty acyls (FAs), glycerolipids (GLs), glycerophospholipids (GPs), sphingolipids (SPs), and sterols (STs) for conversion. For SwissLipids and HMDB, almost the complete selection could be converted into the new nomenclature (Table 3). For LIPID MAPS, about 20% of the lipid names was not recognized, since they are not lipids of confirmed biological origin, contain only trivial names, or simply could not yet be described by the shorthand notation. However, for the SwissLipids and HMDB databases, more than 97% of their lipid names can be converted (Table 3).

**Table 3:** Number of Parsed Lipids per Database: All Database Snapshots Were Acquired in July 2021

	LIPID MAPS	SwissLipids	HMDB
total no. of lipids	45 552	777 956	90 688
total no. of FA, GL, GP, SP, and ST	35 556	777 956	87 775
no. of converted FA, GL, GP, SP, and ST by Goslin 2.0	29 098 (81.8%)	771 287 (99.1%)	85 179 (97.0%)

We further compared Goslin 2.0 to Goslin 1.1.2, RefMet, and LipidLynxX 0.9.24 on a selection of data sets sourced from public data repositories and the literature to assess their speed and percentage of converted lipid names in realistic data conversion and normalization scenarios (see Table S2). On average, Goslin 2.0 was slightly faster than Goslin 1.1.2 (avg. of 2.34 s vs 2.45 s), while being on par with RefMet (avg. of 2.18 s). LipidLynxX was the slowest one (avg. of 79.05 s). Concerning the rate of converted lipid names, Goslin 2.0 outperformed the other tools with an average percentage of 84.11. However, for Goslin, this number only contains lipids that are valid following at least one of the supported grammars. Otherwise, Goslin will not attempt to convert them. Higher percentages for RefMet and LipidLynxX are attributable to a number of questionable conversions (see Table S3). We provide an overview of the specific features supported by Goslin 2.0, Refmet, and LipidLynxX in Table S4.

## Discussion and Conclusion

For standardization of lipid annotation, Goslin 2.0 supports the newest shorthand nomenclature and provides new features such as parsing of systematic fatty acyl chain names or computation of chemical sum formulas, molecular, and adduct masses. To the best of our knowledge, Goslin 2.0 is the first available tool that recognizes nested and recursive patterns problem-free within lipid names. In contrast to error-prone systems using regular expressions, it is very robust against incorrect lipid name descriptions, a crucial feature in automated analysis of very large data sets. Lipid names that do not follow the nomenclature are reported to the user instead of being passed with incorrect annotations. Goslin 2.0 is designed to be a real-time module within pipelines for lipidomics analyses. A key feature is its performance as all implementations can convert a regular-sized list of lipid names into the new nomenclature (including the computation of the sum formulas) in less than a second on current standard computers. It is highly suited to streamline computational lipidomics workflows for true high-throughput analytical experiments. The schematic diagram of the Goslin class model can be found in Figure S1. The Goslin implementations are capable of parsing the IUPAC-IUB systematic lipid names of free fatty acyl chains. Lipid databases such as LIPID MAPS, SwissLipids, or HMDB can automatically update their lipid name entries using our libraries.

Tools like LipidCreator^17^ already profit from the updated version of Goslin due to its straightforward implementation into other standalone tools. It can serve as a cornerstone of standardization in the field of lipidomics^18^ but is still open for updates. All Goslin 2.0 implementations are freely available at https://github.com/lifs-tools/goslin under the terms of liberal open source licenses.

## Supporting Information Available

List of supported lipid classes; comparison between different converters on lipid datasets; and class diagram of the object model used by all Goslin implementations (PDF)
