# Supplementary material for: Goslin 2.0 Implements the Recent Lipid Shorthand Nomenclature for MS-Derived Lipid Structures
Source: Anal Chem. 2022 Apr 11;94(16):6097–101. doi: 10.1021/acs.analchem.1c05430 (PMC9047418; doi:10.1021/acs.analchem.1c05430)
Supplement: Supplementary file 1 — ac1c05430_si_001.pdf [file ac1c05430_si_001.pdf]

Goslin 2.0 implements the recent lipid shorthand nomenclature for MS-derived lipid structures - Supplementary

Dominik Kopczynski[1], Nils Hoffmann[2,3], Bing Peng[4], Gerhard Liebisch[5], Friedrich Spener[6], Robert Ahrends[1,*]

[1] Institute of Analytical Chemistry, University of Vienna, 1090 Vienna, Austria

[2] Center for Biotechnology (CeBiTec), Bielefeld University, 33594 Bielefeld, Germany

[3] Now at: Forschungszentrum Jülich, Institute of Bio- and Geosciences, Computational Metagenomics (IBG-5), 33594 Bielefeld, Germany

[4] Division of Rheumatology, Department of Medicine, Solna, Karolinska Institutet and Karolinska University Hospital, 17176 Stockholm, Sweden

[5] Institute of Clinical Chemistry and Laboratory Medicine, Regensburg University Hospital, 93053 Regensburg, Germany

[6] Department of Molecular Biosciences, University of Graz, 8010 Graz, Austria; Division of Molecular Biology and Biochemistry, Gottfried Schatz Research Center, Medical University of Graz, 8036 Graz, Austria

[*] *Corresponding author. Contact: robert.ahrends@univie.ac.at*

Table of Contents

1. List of supported lipid classes....................2
2. Comparison between different converters on lipid datasets....................10
3. Class diagram of the object model used by all Goslin implementations....................13

1. List of supported lipid classes

Lipid name	Lipid category	Lipid description
10-HDoHE	Fatty Acyls [FA]	Other Docosanoids [FA0400]
11-HDoHE	Fatty Acyls [FA]	Other Docosanoids [FA0400]
11-HETE	Fatty Acyls [FA]	Hydroxy/hydroperoxyeicosatetraenoic acids [FA0306]
11,12-DHET	Fatty Acyls [FA]	Hydroxy/hydroperoxyeicosatetraenoic acids [FA0306]
11(12)-EET	Fatty Acyls [FA]	Epoxyeicosatrienoic acids [FA0308]
12-HEPE	Fatty Acyls [FA]	Hydroxy/hydroperoxyeicosapentaenoic acids [FA0307]
12-HETE	Fatty Acyls [FA]	Hydroxy/hydroperoxyeicosatetraenoic acids [FA0306]
12-HHTrE	Fatty Acyls [FA]	Hydroxy/hydroperoxyeicosatrienoic acids [FA0305]
12-OxoETE	Fatty Acyls [FA]	Fatty acids and conjugates [FA01]
12(13)-EpOME	Fatty Acyls [FA]	Other Octadecanoids [FA0200]
13-HODE	Fatty Acyls [FA]	Other Octadecanoids [FA0200]
13-HOTrE	Fatty Acyls [FA]	Other Octadecanoids [FA0200]
14,15-DHET	Fatty Acyls [FA]	Hydroxy/hydroperoxyeicosatetraenoic acids [FA0306]
14(15)-EET	Fatty Acyls [FA]	Epoxyeicosatrienoic acids [FA0308]
14(15)-EpETE	Fatty Acyls [FA]	Other Eicosanoids [FA0300]
15-HEPE	Fatty Acyls [FA]	Hydroxy/hydroperoxyeicosapentaenoic acids [FA0307]
15-HETE	Fatty Acyls [FA]	Hydroxy/hydroperoxyeicosatetraenoic acids [FA0306]
15d-PGJ2	Fatty Acyls [FA]	Prostaglandins [FA0301]
16-HDoHE	Fatty Acyls [FA]	Other Docosanoids [FA0400]
16-HETE	Fatty Acyls [FA]	Hydroxy/hydroperoxyeicosatetraenoic acids [FA0306]
18-HEPE	Fatty Acyls [FA]	Hydroxy/hydroperoxyeicosapentaenoic acids [FA0307]
5-HEPE	Fatty Acyls [FA]	Hydroxy/hydroperoxyeicosapentaenoic acids [FA0307]
5-HETE	Fatty Acyls [FA]	Hydroxy/hydroperoxyeicosatetraenoic acids [FA0306]
5-HpETE	Fatty Acyls [FA]	Hydroxy/hydroperoxyeicosatetraenoic acids [FA0306]
5-OxoETE	Fatty Acyls [FA]	Fatty acids and conjugates [FA01]
5,12-DiHETE	Fatty Acyls [FA]	Hydroxy/hydroperoxyeicosatetraenoic acids [FA0306]
5,6-DiHETE	Fatty Acyls [FA]	Hydroxy/hydroperoxyeicosatetraenoic acids [FA0306]
5,6,15-LXA4	Fatty Acyls [FA]	Lipoxins [FA0304]
5(6)-EET	Fatty Acyls [FA]	Epoxyeicosatrienoic acids [FA0308]
8-HDoHE	Fatty Acyls [FA]	Other Docosanoids [FA0400]
8-HETE	Fatty Acyls [FA]	Hydroxy/hydroperoxyeicosatetraenoic acids [FA0306]
8,9-DHET	Fatty Acyls [FA]	Hydroxy/hydroperoxyeicosatetraenoic acids [FA0306]
8(9)-EET	Fatty Acyls [FA]	Epoxyeicosatrienoic acids [FA0308]
9-HEPE	Fatty Acyls [FA]	Hydroxy/hydroperoxyeicosapentaenoic acids [FA0307]
9-HETE	Fatty Acyls [FA]	Hydroxy/hydroperoxyeicosatetraenoic acids [FA0306]
9-HODE	Fatty Acyls [FA]	Other Octadecanoids [FA0200]
9-HOTrE	Fatty Acyls [FA]	Other Octadecanoids [FA0200]
9(10)-EpOME	Fatty Acyls [FA]	Other Octadecanoids [FA0200]
AA	Fatty Acyls [FA]	Unsaturated fatty acids [FA0103]
alpha-LA	Fatty Acyls [FA]	Fatty acids and conjugates [FA01]

Lipid name	Lipid category	Lipid description
CAR	Fatty Acyls [FA]	Fatty acyl carnitines [FA0707]
CoA	Fatty Acyls [FA]	Fatty acyl CoAs [FA0705]
DHA	Fatty Acyls [FA]	Unsaturated fatty acids [FA0103]
EPA	Fatty Acyls [FA]	Unsaturated fatty acids [FA0103]
FA	Fatty Acyls [FA]	Fatty acids and conjugates [FA01]
FAHFA	Fatty Acyls [FA]	Wax monoesters [FA0701]
FAL	Fatty Acyls [FA]	Fatty aldehydes [FA06]
FOH	Fatty Acyls [FA]	Fatty alcohols [FA05]
GP-NAE	Fatty Acyls [FA]	Glycerophosphoethanolamine
HC	Fatty Acyls [FA]	Hydrocarbons [FA11]
Linoleic acid	Fatty Acyls [FA]	Unsaturated fatty acids [FA0103]
LTB4	Fatty Acyls [FA]	Leukotrienes [FA0302]
LTC4	Fatty Acyls [FA]	Eicosanoid derivatives
LTD4	Fatty Acyls [FA]	Leukotrienes [FA0302]
Maresin 1	Fatty Acyls [FA]	Maresins [FA0405]
NA	Fatty Acyls [FA]	Fatty amides
NAE	Fatty Acyls [FA]	Fatty amides
NAT	Fatty Acyls [FA]	N-acyl amines [FA0802]
Palmitic acid	Fatty Acyls [FA]	Straight chain fatty acids [FA0101]
PGB2	Fatty Acyls [FA]	Prostaglandins [FA0301]
PGD2	Fatty Acyls [FA]	Prostaglandins [FA0301]
PGE2	Fatty Acyls [FA]	Prostaglandins [FA0301]
PGF2alpha	Fatty Acyls [FA]	Prostaglandins [FA0301]
PGI2	Fatty Acyls [FA]	Prostaglandins [FA0301]
Resolvin D1	Fatty Acyls [FA]	Resolvin Ds [FA0403]
Resolvin D2	Fatty Acyls [FA]	Resolvin Ds [FA0403]
Resolvin D3	Fatty Acyls [FA]	Resolvin Ds [FA0403]
Resolvin D5	Fatty Acyls [FA]	Resolvin Ds [FA0403]
tetranor-12-HETE	Fatty Acyls [FA]	Hydroxy/hydroperoxyeicosatetraenoic acids [FA0306]
TXB1	Fatty Acyls [FA]	Thromboxanes [FA0303]
TXB2	Fatty Acyls [FA]	Thromboxanes [FA0303]
TXB3	Fatty Acyls [FA]	Thromboxanes [FA0303]
WD	Fatty Acyls [FA]	Wax diesters [FA0702]
WE	Fatty Acyls [FA]	Fatty esters [FA07]
DG	Glycerolipids [GL]	Diacylglycerols [GL0201]
DGCC	Glycerolipids [GL]	Other Glycerolipids [GL00]
DGDG	Glycerolipids [GL]	Glycosyldiradylglycerols [GL05]
DGMG	Glycerolipids [GL]	Glycosyldiradylglycerols [GL05]
DHDG	Glycerolipids [GL]	Dihexosyldiacylglycerol
Glc-DG	Glycerolipids [GL]	Glycosyldiradylglycerols [GL05]
MG	Glycerolipids [GL]	Monoacylglycerols [GL0101]
MGDG	Glycerolipids [GL]	Glycosyldiacylglycerols [GL0501]
MGMG	Glycerolipids [GL]	Glycosyldiacylglycerols [GL0501]

**Lipid name**	**Lipid category**	**Lipid description**
MHDG	Glycerolipids [GL]	Monohexosyldiacylglycerol
PE-GlcDG	Glycerolipids [GL]	Glycosylalkylacylglycerols [GL0502]
SQDG	Glycerolipids [GL]	Glycosyldiradylglycerols [GL05]
SQMG	Glycerolipids [GL]	Glycosylmonoacylglycerols [GL0401]
TG	Glycerolipids [GL]	Triacylglycerols [GL0301]
6-Ac-Glc-GP	Glycerophospholipids [GP]	Glycosylglycerophospholipids [GP14]
BMP	Glycerophospholipids [GP]	Monoacylglycerophosphomonoradylglycerols [GP0410]
CDPDAG	Glycerophospholipids [GP]	CDP-diacylglycerols [GP1301]
CL	Glycerophospholipids [GP]	Glycerophosphoglycerophosphoglycerols [GP12]
CPA	Glycerophospholipids [GP]	Other Glycerophospholipids [GP00]
DLCL	Glycerophospholipids [GP]	Glycerophosphoglycerophosphoglycerols [GP12]
DMPE	Glycerophospholipids [GP]	Dimethylphosphatidylethanolamine
Glc-GP	Glycerophospholipids [GP]	Diacylglycosylglycerophospholipids [GP1401]
LCDPDAG	Glycerophospholipids [GP]	Lyso-CDP-diacylglycerol
LCL	Glycerophospholipids [GP]	Glycerophosphoglycerophosphoglycerols [GP12]
LDMPE	Glycerophospholipids [GP]	Lysodimethylphosphatidylethanolamine
LMMPE	Glycerophospholipids [GP]	Lysomonomethylphosphatidylethanolamine
LPA	Glycerophospholipids [GP]	Monoacylglycerophosphates [GP1005]
LPC	Glycerophospholipids [GP]	Monoacylglycerophosphocholines [GP0105]
LPE	Glycerophospholipids [GP]	Monoacylglycerophosphoethanolamines [GP0205]
LPE-N	Glycerophospholipids [GP]	N-acyl/alkyl PE
LPG	Glycerophospholipids [GP]	Monoacylglycerophosphoglycerols [GP0405]
LPI	Glycerophospholipids [GP]	Monoacylglycerophosphoinositols [GP0605]
LPIM1	Glycerophospholipids [GP]	Monoacylglycerophosphoinositolglycans [GP1504]
LPIM2	Glycerophospholipids [GP]	Monoacylglycerophosphoinositolglycans [GP1504]
LPIM3	Glycerophospholipids [GP]	Monoacylglycerophosphoinositolglycans [GP1504]
LPIM4	Glycerophospholipids [GP]	Monoacylglycerophosphoinositolglycans [GP1504]
LPIM5	Glycerophospholipids [GP]	Monoacylglycerophosphoinositolglycans [GP1504]
LPIM6	Glycerophospholipids [GP]	Monoacylglycerophosphoinositolglycans [GP1504]
LPIMIP	Glycerophospholipids [GP]	Lysophosphatidylinositol- mannosideinositolphosphate
LPIN	Glycerophospholipids [GP]	Lysophosphatidylinositol-glucosamine
LPS	Glycerophospholipids [GP]	Monoacylglycerophosphoserines [GP0305]
MMPE	Glycerophospholipids [GP]	Monomethylphosphatidylethanolamine
NAPE	Glycerophospholipids [GP]	Diacylglycerophosphoethanolamines [GP0201]
PA	Glycerophospholipids [GP]	Glycerophosphates [GP10]
PC	Glycerophospholipids [GP]	Glycerophosphocholines [GP01]
PE	Glycerophospholipids [GP]	Glycerophosphoethanolamines [GP02]
PE-N	Glycerophospholipids [GP]	N-acyl/alkyl PE
PE-NMe	Glycerophospholipids [GP]	Diacylglycerophosphoethanolamines [GP0201]
PE-NMe2	Glycerophospholipids [GP]	Diacylglycerophosphoethanolamines [GP0201]
PEt	Glycerophospholipids [GP]	Glycerophosphoethanolamines [GP02]
PG	Glycerophospholipids [GP]	Glycerophosphoglycerols [GP04]
PGP	Glycerophospholipids [GP]	Glycerophosphoglycerophosphates [GP05]

Lipid name	Lipid category	Lipid description
PI	Glycerophospholipids [GP]	Glycerophosphoinositols [GP06]
PIM1	Glycerophospholipids [GP]	Glycerophosphoinositolglycans [GP15]
PIM2	Glycerophospholipids [GP]	Glycerophosphoinositolglycans [GP15]
PIM3	Glycerophospholipids [GP]	Glycerophosphoinositolglycans [GP15]
PIM4	Glycerophospholipids [GP]	Glycerophosphoinositolglycans [GP15]
PIM5	Glycerophospholipids [GP]	Glycerophosphoinositolglycans [GP15]
PIM6	Glycerophospholipids [GP]	Glycerophosphoinositolglycans [GP15]
PIMIP	Glycerophospholipids [GP]	Phosphatidylinositol mannoside inositol phosphate
PIP	Glycerophospholipids [GP]	Diacylglycerophosphoinositol monophosphates [GP0701]
PIP(3')	Glycerophospholipids [GP]	Diacylglycerophosphoinositol monophosphates [GP0701]
PIP(4')	Glycerophospholipids [GP]	Diacylglycerophosphoinositol monophosphates [GP0701]
PIP(5')	Glycerophospholipids [GP]	Diacylglycerophosphoinositol monophosphates [GP0701]
PIP2	Glycerophospholipids [GP]	Diacylglycerophosphoinositol bisphosphates [GP0801]
PIP2(3',4')	Glycerophospholipids [GP]	Diacylglycerophosphoinositol bisphosphates [GP0801]
PIP2(3',5')	Glycerophospholipids [GP]	Diacylglycerophosphoinositol bisphosphates [GP0801]
PIP2(4',5')	Glycerophospholipids [GP]	Diacylglycerophosphoinositol bisphosphates [GP0801]
PIP3	Glycerophospholipids [GP]	Diacylglycerophosphoinositol trisphosphates [GP0901]
PIP3(3',4',5')	Glycerophospholipids [GP]	Diacylglycerophosphoinositol trisphosphates [GP0901]
PnC	Glycerophospholipids [GP]	Glycerophosphonocholines [GP16]
PnE	Glycerophospholipids [GP]	Glycerophosphoinositolglycans [GP15]
PPA	Glycerophospholipids [GP]	Diacylglyceropyrophosphates [GP1101]
PS	Glycerophospholipids [GP]	Glycerophosphoserines [GP03]
PS-N	Glycerophospholipids [GP]	N-acyl/alkyl PS
PS-NAc	Glycerophospholipids [GP]	Diacylglycerophosphoserines [GP0301]
PT	Glycerophospholipids [GP]	Other Glycerophospholipids [GP00]
SLBPA	Glycerophospholipids [GP]	Diacylglycerophosphomonoradylglycerols [GP0409]
ANACARD	Polyketides [PK]	Anacardic acids and derivatives [PK1504]
CATECHOL	Polyketides [PK]	Alkyl catechols and derivatives [PK1502]
PHENOL	Polyketides [PK]	Alkyl phenols and derivatives [PK1501]
RESORCINOL	Polyketides [PK]	Alkyl resorcinols and derivatives [PK1503]
AC2SGL	Saccharolipids [SL]	Acyltrehaloses [SL03]
DAT	Saccharolipids [SL]	Acyltrehaloses [SL03]
PAT16	Saccharolipids [SL]	Acyltrehaloses [SL03]
PAT18	Saccharolipids [SL]	Acyltrehaloses [SL03]
(3'-sulfo)LacCer	Sphingolipids [SP]	Glycosphingolipids
(Fuc)iGb3Cer	Sphingolipids [SP]	Glycosphingolipids
1-O-behenoyl-Cer	Sphingolipids [SP]	Acylceramides [SP0204]
1-O-carboceroyl-Cer	Sphingolipids [SP]	Acylceramides [SP0204]
1-O-cerotoyl-Cer	Sphingolipids [SP]	Acylceramides [SP0204]
1-O-eicosanoyl-Cer	Sphingolipids [SP]	Acylceramides [SP0204]

Lipid name	Lipid category	Lipid description
1-O-lignoceroyl-Cer	Sphingolipids [SP]	Acylceramides [SP0204]
1-O-myristoyl-Cer	Sphingolipids [SP]	Acylceramides [SP0204]
1-O-palmitoyl-Cer	Sphingolipids [SP]	Acylceramides [SP0204]
1-O-stearoyl-Cer	Sphingolipids [SP]	Acylceramides [SP0204]
1-O-tricosanoyl-Cer	Sphingolipids [SP]	Acylceramides [SP0204]
Ac-O-9-GD1a	Sphingolipids [SP]	Ganglioside
Ac-O-9-GT1b	Sphingolipids [SP]	Ganglioside
Ac-O-9-GT3	Sphingolipids [SP]	Ganglioside
ACer	Sphingolipids [SP]	Acylceramide [SP0204]
Branched-Forssman	Sphingolipids [SP]	Glycosphingolipids
Cer	Sphingolipids [SP]	Ceramides [SP02]
CerP	Sphingolipids [SP]	Ceramide 1-phosphates [SP0205]
DSGG	Sphingolipids [SP]	Glycosphingolipids
EPC	Sphingolipids [SP]	Ceramide phosphoethanolamines [SP0302]
FMC-5	Sphingolipids [SP]	Simple Glc series [SP0501]
FMC-6	Sphingolipids [SP]	Neutral glycosphingolipids [SP05]
Forssman	Sphingolipids [SP]	Glycosphingolipids
Fuc-Branched-Forssman	Sphingolipids [SP]	Glycosphingolipids
Fuc-GA1	Sphingolipids [SP]	Ganglioside
Fuc-GD1b	Sphingolipids [SP]	Ganglioside
Fuc-GM1	Sphingolipids [SP]	Ganglioside
Fuc-GM1(NeuGc)	Sphingolipids [SP]	Ganglioside
Fuc-iGb3Cer	Sphingolipids [SP]	Glycosphingolipids
Fuc(Gal)-GM1	Sphingolipids [SP]	Acidic glycosphingolipids [SP06]
Fuc(Gal)Gal-iGb4Cer	Sphingolipids [SP]	Glycosphingolipids
FucGalGb3Cer	Sphingolipids [SP]	Glycosphingolipids
GA1	Sphingolipids [SP]	Glycosphingolipids
GA2	Sphingolipids [SP]	Glycosphingolipids
Gal-GD1b	Sphingolipids [SP]	Ganglioside
Gal-iGb4Cer	Sphingolipids [SP]	Glycosphingolipids
Gal(Fuc)-GA1	Sphingolipids [SP]	Ganglioside
Gal(Fuc)-GD1b	Sphingolipids [SP]	Ganglioside
GalCer	Sphingolipids [SP]	Neutral glycosphingolipids [SP05]
GalGal-GD1b	Sphingolipids [SP]	Ganglioside
GalGalGalGb3Cer	Sphingolipids [SP]	Glycosphingolipids
GalGalGb3Cer	Sphingolipids [SP]	Glycosphingolipids
GalGalNAc-GM1b(NeuGc)	Sphingolipids [SP]	Ganglioside
GalGb3Cer	Sphingolipids [SP]	Glycosphingolipids
GalGb4Cer	Sphingolipids [SP]	Glycosphingolipids
GalGlcNAc-GalGb4Cer	Sphingolipids [SP]	Glycosphingolipids

Lipid name	Lipid category	Lipid description
GalNAc-GD1a	Sphingolipids [SP]	Ganglioside
GalNAc-GD1a(NeuAc/NeuGc)	Sphingolipids [SP]	Ganglioside
GalNAc-GD1a(NeuGc/NeuAc)	Sphingolipids [SP]	Ganglioside
GalNAc-GM1	Sphingolipids [SP]	Ganglioside
GalNAc-GM1b	Sphingolipids [SP]	Ganglioside
GalNAc-GM1b(NeuGc)	Sphingolipids [SP]	Ganglioside
GalNAcGal(Fuc)-GA1	Sphingolipids [SP]	Ganglioside
GalNAcGalGb3Cer	Sphingolipids [SP]	Glycosphingolipids
GB3	Sphingolipids [SP]	Neutral glycosphingolipids [SP05]
GB4	Sphingolipids [SP]	Neutral glycosphingolipids [SP05]
GD1	Sphingolipids [SP]	Glycosphingolipids
GD1a	Sphingolipids [SP]	Ganglioside
GD1a alpha	Sphingolipids [SP]	Ganglioside
GD1a(NeuAc/NeuGc)	Sphingolipids [SP]	Ganglioside
GD1a(NeuGc/NeuAc)	Sphingolipids [SP]	Ganglioside
GD1a(NeuGc/NeuGc)	Sphingolipids [SP]	Ganglioside
GD1b	Sphingolipids [SP]	Ganglioside
GD1c	Sphingolipids [SP]	Ganglioside
GD1c(NeuGc/NeuGc)	Sphingolipids [SP]	Ganglioside
GD2	Sphingolipids [SP]	Ganglioside
GD3	Sphingolipids [SP]	Ganglioside
GlcCer	Sphingolipids [SP]	Neutral glycosphingolipids [SP05]
GlcNAc-GalGb4Cer	Sphingolipids [SP]	Globoside
GlcNAcGb3Cer	Sphingolipids [SP]	Globoside
Globo-A	Sphingolipids [SP]	Globoside
Globo-B	Sphingolipids [SP]	Globoside
Globo-H	Sphingolipids [SP]	Globoside
Globo-Lex-9	Sphingolipids [SP]	Globoside
GM1	Sphingolipids [SP]	Ganglioside
GM1 alpha	Sphingolipids [SP]	Ganglioside
GM1(NeuGc)	Sphingolipids [SP]	Ganglioside
GM1b	Sphingolipids [SP]	Ganglioside
GM1b(NeuGc)	Sphingolipids [SP]	Ganglioside
GM2	Sphingolipids [SP]	Ganglioside
GM2(NeuGc)	Sphingolipids [SP]	Ganglioside
GM3	Sphingolipids [SP]	Ganglioside
GM4	Sphingolipids [SP]	Ganglioside
GP1	Sphingolipids [SP]	Ganglioside
GP1c	Sphingolipids [SP]	Ganglioside
GP1c alpha	Sphingolipids [SP]	Ganglioside

Lipid name	Lipid category	Lipid description
GQ1	Sphingolipids [SP]	Ganglioside
GQ1b	Sphingolipids [SP]	Ganglioside
GQ1b alpha	Sphingolipids [SP]	Ganglioside
GQ1c	Sphingolipids [SP]	Ganglioside
GT1	Sphingolipids [SP]	Ganglioside
GT1a	Sphingolipids [SP]	Ganglioside
GT1a alpha	Sphingolipids [SP]	Ganglioside
GT1b	Sphingolipids [SP]	Ganglioside
GT1b alpha	Sphingolipids [SP]	Ganglioside
GT1b alpha(NeuGc)	Sphingolipids [SP]	Ganglioside
GT1c	Sphingolipids [SP]	Ganglioside
GT2	Sphingolipids [SP]	Ganglioside
GT3	Sphingolipids [SP]	Ganglioside
Hex2Cer	Sphingolipids [SP]	Neutral glycosphingolipids [SP05]
Hex3Cer	Sphingolipids [SP]	Neutral glycosphingolipids [SP05]
HexCer	Sphingolipids [SP]	Neutral glycosphingolipids [SP05]
i-Forssman	Sphingolipids [SP]	Isogloboside
iGb3Cer	Sphingolipids [SP]	Isogloboside
iGb4Cer	Sphingolipids [SP]	Isogloboside
IPC	Sphingolipids [SP]	Ceramide phosphoinositols [SP0303]
LacCer	Sphingolipids [SP]	Neutral glycosphingolipids [SP05]
Lex-GM1	Sphingolipids [SP]	Ganglioside
LHexCer	Sphingolipids [SP]	Lysohexosylceramide
LIPC	Sphingolipids [SP]	Ceramide phosphoinositols [SP0303]
LSM	Sphingolipids [SP]	Ceramides [SP02]
M(IP)2C	Sphingolipids [SP]	Phosphosphingolipids [SP03]
MIPC	Sphingolipids [SP]	Phosphosphingolipids [SP03]
MSGG	Sphingolipids [SP]	Globoside
NeuAc(alpha2-6)-MSGG	Sphingolipids [SP]	Globoside
NeuAc(alpha2-8)-MSGG	Sphingolipids [SP]	Globoside
NeuAcGal-iGb4Cer	Sphingolipids [SP]	Isogloboside
NeuGc-GalGb4Cer	Sphingolipids [SP]	Globoside
NeuGc-LacNAc-GM1(NeuGc)	Sphingolipids [SP]	Ganglioside
NeuGcNeuGc-GalGb4Cer	Sphingolipids [SP]	Ganglioside
NOR1	Sphingolipids [SP]	Globoside
NOR2	Sphingolipids [SP]	Globoside
NORint	Sphingolipids [SP]	Globoside
Para-Forssman	Sphingolipids [SP]	Globoside
SB1a	Sphingolipids [SP]	Globoside
SHex2Cer	Sphingolipids [SP]	Dihexosylsulfatide

Lipid name	Lipid category	Lipid description
SHexCer	Sphingolipids [SP]	Sulfoglycosphingolipids (sulfatides) [SP0602]
SM	Sphingolipids [SP]	Ceramide phosphocholines (sphingomyelins) [SP0301]
SM1a	Sphingolipids [SP]	Globoside
SM1b	Sphingolipids [SP]	Globoside
SO3-Gal-iGb4Cer	Sphingolipids [SP]	Isogloboside
SO3-GalGb4Cer	Sphingolipids [SP]	Ganglioside
SO3-Gb4Cer	Sphingolipids [SP]	Ganglioside
SO3-GM1(NeuGc)	Sphingolipids [SP]	Ganglioside
SO3-iGb4Cer	Sphingolipids [SP]	Isogloboside
SPB	Sphingolipids [SP]	Sphingoid base homologs and variants [SP0104]
SPBP	Sphingolipids [SP]	Sphingoid base 1-phosphates [SP0105]
SulfoGalCer	Sphingolipids [SP]	Sulfogalactosyl ceramide
ASG	Sterol Lipids [ST]	Acylated steryl glucosides
BA	Sterol Lipids [ST]	Bile acids and derivatives [ST04]
SE 27:1	Sterol Lipids [ST]	Sterol esters [ST0102]
SE 27:2	Sterol Lipids [ST]	Sterol esters [ST0102]
SE 28:2	Sterol Lipids [ST]	Sterol esters [ST0102]
SE 28:3	Sterol Lipids [ST]	Sterol esters [ST0102]
SE 29:2	Sterol Lipids [ST]	Sterol esters [ST0102]
SE 30:2	Sterol Lipids [ST]	Sterol esters [ST0102]
SG	Sterol Lipids [ST]	Sterylglycosides
ST 27:1;1	Sterol Lipids [ST]	Cholesterol and derivatives [ST0101]
ST 27:2;1	Sterol Lipids [ST]	Cholesterol and derivatives [ST0101]
ST 28:1;1	Sterol Lipids [ST]	Cholesterol and derivatives [ST0101]
ST 28:2;1	Sterol Lipids [ST]	Ergosterols and C24-methyl derivatives [ST0103]
ST 28:3;1	Sterol Lipids [ST]	Ergosterols and C24-methyl derivatives [ST0103]
ST 29:1;1	Sterol Lipids [ST]	Cholesterol and derivatives [ST0101]
ST 29:2;1	Sterol Lipids [ST]	Stigmasterols and C24-ethyl derivatives [ST0104]
ST 30:2;1	Sterol Lipids [ST]	Cholesterol and derivatives [ST0101]

Table S1: All supported lipid classes by Goslin 2.0. The full table is available from our GitHub repository: https://github.com/lifs-tools/goslin/blob/master/lipid-list.csv. The abbreviations are either adopted from the latest shorthand nomenclature by Liebisch et al. (2020)[1] or derived from databases such as LIPID MAPS, SwissLipids, or HMDB.

1 Liebisch, Gerhard, et al. "Update on LIPID MAPS classification, nomenclature, and shorthand notation for MS-derived lipid structures." Journal of lipid research 61.12 (2020): 1539-1555.

2. Comparison between different converters on lipid datasets

Dataset (number of species)	*Goslin 2.0*		*Goslin 1.1.2*		*RefMet*		*LipidLynxX 0.9.24*	
	time (s)	*conv %*	*time (s)*	*conv %*	*time (s)*	*conv %*	*time (s)*	*conv %*
ST001710 (176)	1.3	82.3	1.3	82.3	1.3	95.4	24.0	100
ST001942 (206)	1.1	63.1	1.3	63.1	1.3	63.1	19.1	100
ST001950 (584)	1.6	56.8	1.4	56.8	1.9	92.9	61.1	88.6
ST002070 (259)	1.0	64.4	1.3	59.8	1.4	99.6	23.6	94.9
ST002079 (3464)	7.7	75.7	6.7	75.7	5.9	98.4	354	97.0
PMID: 22848500 (319)	1.2	55.1	1.3	54.8	1.4	89.9	20.8	90.2
PMID: 25794437 (223)	0.9	95.9	1.1	94.6	1.3	82	14.9	84.3
PMID: 27053105 (463)	1.2	94.8	1.0	65.0	1.5	84.6	31.3	56.8
PMID: 27756783 (200)	0.7	100	0.6	100	1.2	100	16.5	99.5
PMID: 29058722 (237)	1.1	89.4	1.0	89.4	0.9	89.4	22.1	100
LipidCreator dataset (1757)	3.1	100	2.9	97.9	3.5	45.3	144.9	71.4
LIPID MAPS (1000)	5.1	100	3.4	100	2.3	89.3	131.2	90.8
FA chains shorthand (1023)	1.8	100	1.9	0	2.5	0	82.0	99.3
FA chains IUPAC (1956)	5.0	100	9.1	0	4.1	20.5	161.2	3.3
Average	2.34	84.11	2.45	67.1	2.18	75.03	79.05	84.01

Table S2: Tools Goslin 2.0, Goslin 1.1.2, RefMet and LipidLynxX 0.9.24 were compared with each other in terms of computational time in seconds and rate of converted lipids in percent. We tested 10 datasets sourced from the literature and from Metabolomics Workbench. Datasets beginning with ST are Metabolomics Workbench IDs, datasets beginning with PMID are PubMed IDs. The number in brackets indicates the number of lipids in each set. In general, it is observable that RefMet and LipidLynxX convert more unusual lipid names such as "LPC(18:1).1" or "DG(48:2)_or_DG(24:2_24:0)". Obviously, these lipid names contain additional information, like an index of elution or several different lipid names in one line separated with different separators. These lipid names do not follow any defined nomenclature and thus are critical to consider especially when multiple names are reported at once. It should be noted, that both RefMet and LipidLynxX convert at most one of multiple lipid names for cases like the example above. When cleaning these lipid names by, e.g., splitting multiple lipid names into several lines, Goslin 2.0 is reaching comparable conversion results as RefMet. Although LipidLynxX has the highest conversion rate, it also has the highest error rate with questionable conversions. An additional human review step is necessary after conversion which makes the usage in automated workflows inapplicable.

	LipidLynxX			*RefMet*	
Input lipid	*Converted lipid*	*Issue*	*Input lipid*	*Converted lipid*	*Issue*
DG 18:2/-/18:2	DG 18:2	Illegal input, conversion without warnings	DG(36:1)>DG(18:0_18:1_0:0)	-	No conversion, but next line entry is converted
Cer 17:0	Cer 17:0	Does not conform to nomenclature	DG(36:2)>DG(18:1_18:1_0:0)	DG 18:1_18:1	Second name is converted, first is ignored
Cer 18:0	Cer 18:1;2/18:0	Extra information added	PE 18:1(5E)/16:0	PE 18:1(5E)/16:0	Correct conversion, but see next line
HexCer(d42:0-OH)	Cer 0:0	Improper conversion, no warning	PE 18:0/16:1(4E)	-	No conversion, inconsistent to conversion above
FA(18:5)(Ke)	FA 18:5	Ignoring additional functional groups			
FA 10:1(2E);10OMe;10oxo	FA 10:1(2E)	Ignoring additional functional groups			
FA 18:0;9Ep	FA 18:0;9	Ignoring additional functional groups			
GM3 18:1;2/24:1	EXCEPTIONS 18:1;2/24:1	Exception message part of lipid name			

Table S3: Examples of questionable or inconsistent conversions of LipidLynxX and RefMet. For each tool, we list the input lipid and the converted lipid together with the issue inherent in the conversion. Goslin 2.0 would either convert lipid names that conform to one of its supported grammars or would stop / skip the conversion and report the issue immediately.

Feature	*Goslin 2.0*	*RefMet*	*LipidLynxX*
Conversion of lipid names	yes	yes	yes
Download result table	yes	yes	yes
Provides lipid class	yes	yes	no
Provides lipid category	yes	yes	no
Provides exact mass	**yes**	yes	no
Provides sum formula	**yes**	yes	no
Provides links to further databases	yes	yes	no
Supports updated shorthand nomenclature	**yes**	partially	no
Provides information on total no. of Cs, double bonds, etc. on bulk level	yes	yes	no
Provides information on no. of Cs, double bonds, etc. on fatty acyl / long chain base level	yes	no	no
Provides lipid hierarchy level (e.g., *sn*-defined, molecular species)	**yes**	no	no
Supports adducts	yes	no	no
Application programming interface (API)	yes	yes	no
Provision in other programming languages	**C++/C#/Java/Pyth-on/R**	no	Python
Open Source code availability	**yes**	no	yes

Table S4: Comparison of different features provided by Goslin 2.0, RefMet, and LipidLynxX (bold main improvements).

3. Class diagram of the object model used by all Goslin implementations

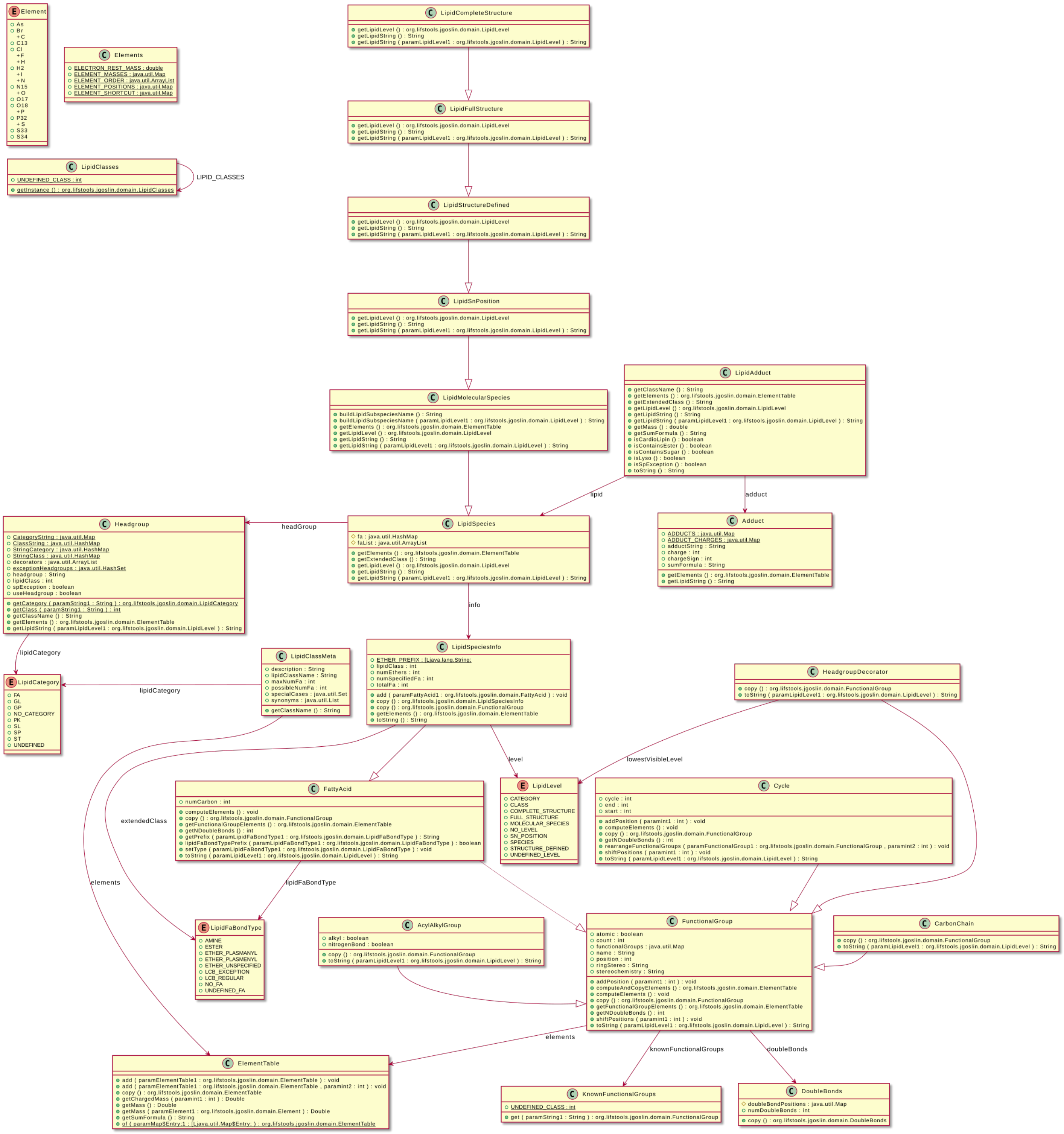

Figure S1: Class diagram of the central Goslin data model and relationships between individual classes. The data model is largely shared between the different programming language implementations. A higher resolution and a lossless vector-graphics version of the class diagram are available from our GitHub repository:
PNG: https://raw.githubusercontent.com/lifs-tools/goslin/master/docs/goslin-class-diagram.png
SVG: https://raw.githubusercontent.com/lifs-tools/goslin/master/docs/goslin-class-diagram.svg
